# Gallbladder Carcinoma in the United States: A Population Based Clinical Outcomes Study Involving 22,343 Patients from the Surveillance, Epidemiology, and End Result Database (1973–2013)

**DOI:** 10.1155/2017/1532835

**Published:** 2017-05-30

**Authors:** Christine S. M. Lau, Aleksander Zywot, Krishnaraj Mahendraraj, Ronald S. Chamberlain

**Affiliations:** ^1^Department of Surgery, Saint Barnabas Medical Center, Livingston, NJ, USA; ^2^School of Medicine, Saint George's University, True Blue, Grenada; ^3^Department of Surgery, Banner MD Anderson Cancer Center, Gilbert, AZ, USA; ^4^Department of Surgery, New Jersey Medical School, Rutgers University, Newark, NJ, USA

## Abstract

**Introduction:**

Gallbladder carcinoma (GBC) is the most common malignancy of the biliary tract and the third most common gastrointestinal tract malignancy. This study examines a large cohort of GBC patients in the United States in an effort to define demographics, clinical, and pathologic features impacting clinical outcomes.

**Methods:**

Demographic and clinical data on 22,343 GBC patients was abstracted from the SEER database (1973–2013).

**Results:**

GBC was presented most often among Caucasian (63.9%) females (70.7%) as poorly or moderately differentiated (42.5% and 38.2%) tumors, with lymph node involvement (88.2%). Surgery alone was the most common treatment modality for GBC patients (55.0%). Combination surgery and radiation (10.6%) achieved significantly longer survival rates compared to surgery alone (4.0 ± 0.2 versus 3.7 ± 0.1 years, *p* = 0.004). Overall mortality was 87.0% and cancer-specific mortality was 75.4%.

**Conclusions:**

GBC is an uncommon malignancy that presents most often among females in their 8th decade of life, with over a third of cases presenting with distant metastasis. The incidence of GBC has doubled in the last decade concurrent with increases in cholecystectomy rates attributable in part to improved histopathological detection, as well as laparoscopic advances and enhanced endoscopic techniques. Surgical resection confers significant survival benefit in GBC patients.

## 1. Introduction

Gallbladder carcinoma (GBC) is the most common malignancy of the biliary tract and third most common gastrointestinal tract malignancy [1, 2]. While a majority of patients are asymptomatic and are diagnosed incidentally following cholecystectomy for gallstones, some patients present with advanced disease with vague abdominal symptoms including abdominal pain and discomfort [2–6]. The incidence of GBC is especially high among South America, affecting 27 per 100,000 people [1]. The high rates of GBC in South America and Asia including Pakistan, Korea, and Japan have been attributed to high rates of cholecystitis and salmonella infection, both of which are known risk factors for GBC [7, 8]. Although gallbladder carcinoma is much less prevalent in North America compared to Asia, it is still associated with an extremely poor prognosis [5, 6].

Given the low rates of GBC in North American, most existing knowledge regarding GBC is derived primarily from studies conducting in South America and Asia. This study examines a large cohort of gallbladder carcinoma patients in the United States (US) in an effort to define the demographics, clinical, and pathologic features impacting clinical outcomes in American GBC patients.

## 2. Methods

Data for the current study was extracted from the Surveillance, Epidemiology, and End Result (SEER) database provided by the National Cancer Institute between 1973 and 2013. SEER Stat software version 8.0.4 was utilized to extract data from 18 SEER registries (Alaska Native Tumor Registry, Arizona Indians, Cherokee Nation, Connecticut, Detroit, Georgia Center for Cancer Statistics, Greater Bay Area Cancer Registry, Greater California, Hawaii, Iowa, Kentucky, Los Angeles, Louisiana, New Jersey, New Mexico, Seattle-Puget Sound, and Utah).

22,343 patients with GBC were identified using the SEER International Classification of Disease for Oncology (ICD-O-3) codes C23.9 [9]. Demographic and clinical data extracted included age, gender, race, tumor grade, lymph node involvement, and type of treatment received (surgery, radiation, both, or unknown/no treatment) [9]. Outcomes examined included mortality and cancer-specific mortality.* Chi*-square test was used to compare categorical data, and Student's *t*-test and analysis of variance (ANOVA) were used for continuous data. Multivariate analysis was performed and odds ratios (OR) were calculated to determine independent factors affecting survival. Long-term actuarial survival between groups was compared using Kaplan Meier analysis. Data was analyzed using IBM SPSS®v23 and statistical significance was accepted at the level of *p* < 0.05 [10].

## 3. Results

### 3.1. Demographic Data

**Figure 1:**
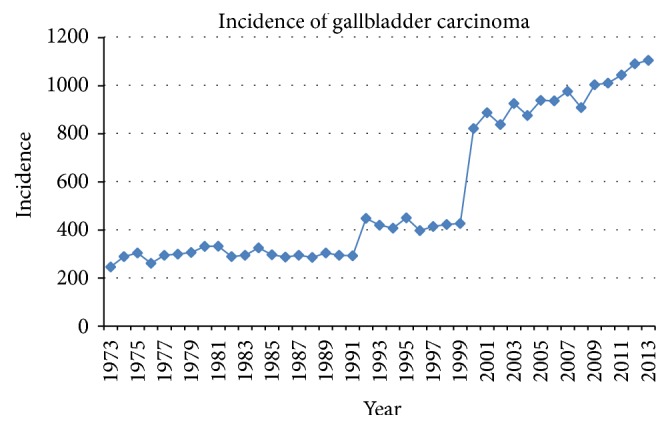
Annual cases of gallbladder carcinoma from the Surveillance, Epidemiology, and End Result (SEER) database (1973–2013).

A total of 22,343 cases of GBC were reported in the SEER database from 1973 to 2013. The number of GBC cases increased from approximately 200 cases per year in the 1970s to >1,000 cases per year after 2010, with a significant spike in 2000 (Table 1 and Figure 1). The average age at diagnosis was 71.2 ± 12.5 years (Table 2). GBC was significantly more common among females (70.7% versus 29.3%), with a female-to-male ratio of 2.41 : 1. A majority of GBC cases occurred among Caucasians (63.9%), followed by Hispanics (16.8%), African Americans (9.2%), and Asian/Pacific Islanders (1.7%). Most of the reported cases occurred in the Pacific Coast (43.0%), followed by the East (29.9%), Northern Plains (18.9%), Southwest (8.0%), and Alaska region (0.2%).

### 3.2. Tumor Characteristics

Most cases of GBC presented as poorly differentiated tumors (42.5%), followed by moderately differentiated (38.2%), well differentiated (15.3%), and undifferentiated (4.0%) tumors. Most patients presented with lymph node involvement (88.2%).

### 3.3. Treatment

**Figure 2:**
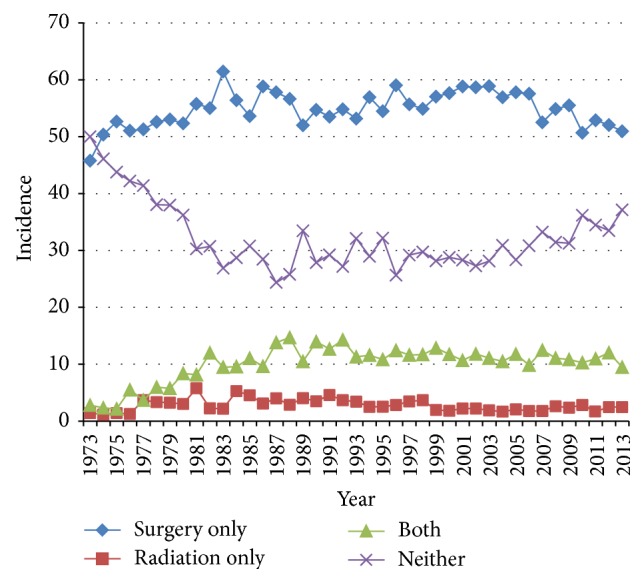
Trends in the treatment modalities utilized for gallbladder carcinoma from the Surveillance, Epidemiology, and End Result (SEER) database (1973–2013).

Surgical resection alone was the most common treatment modality (55.0%). Surgical resection and adjuvant radiation were utilized by 10.6%, while radiation alone was used in 2.6% of patients. 31.8% of patients received neither surgery nor radiation. The number of GBC treated with surgery increased in the 1980s, with a concomitant decrease in patients receiving no treatment (Figure 2).

### 3.4. Outcomes

**Figure 3:**
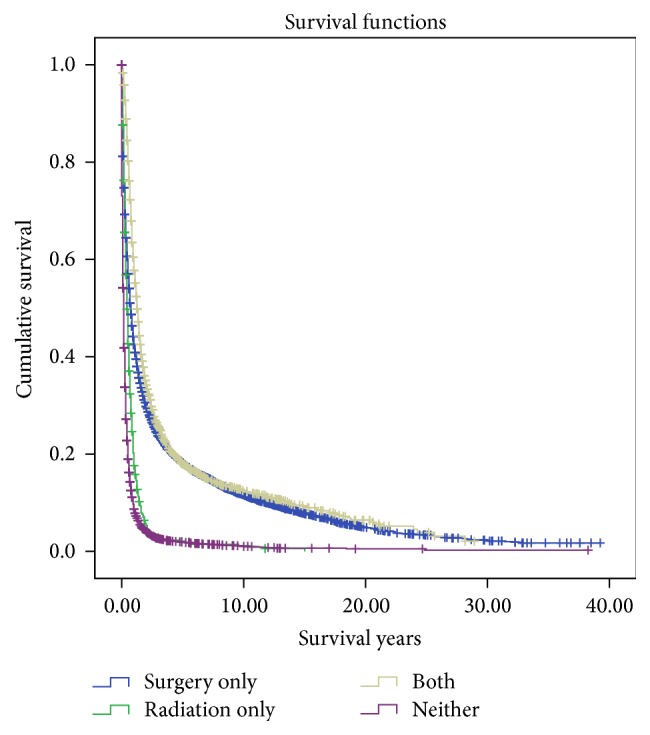
Kaplan Meier curves illustrating actuarial survival for patients with gallbladder carcinoma from the Surveillance, Epidemiology, and End Results database (1973–2013).

Overall survival was 2.72 ± 0.06 years. Surgical resection was associated with significantly improved survival (3.69 ± 0.09 years) compared to patients receiving no treatment (0.62 ± 0.05 years) or radiation alone (0.82 ± 0.08 years) (Table 3). Surgical resection and adjuvant radiation were associated with a slightly longer survival compared to surgical resection alone (4.03 ± 0.18 years versus 3.69 ± 0.09 years, *p* < 0.01). Kaplan Meier estimates also demonstrated prolonged survival for patients receiving surgical resection with or without adjuvant radiation (Figure 3).

When stratified by tumor grade, well differentiated tumors had the longest survival (5.93 ± 0.27 years), followed by moderately differentiated (3.72 ± 0.15 years), poorly differentiated (1.66 ± 0.07 years), and undifferentiated (1.29 ± 0.17 years) tumors.

Overall mortality was 87.0% and cancer-specific mortality was 75.4%. Cumulative survival remained low, and 1-, 2-, and 5-year survival were 34%, 22%, and 13%, respectively.

### 3.5. Multivariate Analysis

Multivariate analysis identified moderately differentiated (OR 1.43; 95% CI, 1.27–1.61), poorly differentiated (OR 3.10; 95% CI, 2.72–3.54), and undifferentiated (OR 3.10; 95% CI, 2.26–4.25) tumors as independently associated with increased mortality, *p* < 0.05. Conversely, surgical resection (OR 0.406; 95% CI, 0.311–0.529) and combined surgery and radiation (OR 0.321; 95% CI, 0.242–0.426) were associated with reduced mortality, *p* < 0.05.

## 4. Discussion

GBC is an aggressive malignancy associated with multiple etiologies and high mortality [1, 8]. Despite being the most common biliary tract malignancy and the fifth most common gastrointestinal cancer, GBC is rare [2]. The overall incidence of GBC worldwide varies greatly from 1.5 per 100,000 in North America to as high as 27 per 100,000 in South America [1].

The large variation in incidence worldwide is due to a combination of exposure to environmental risk factors and heritable genetic traits [1, 3]. The incidence of GBC increases with age, ranging from 1.47 per 100,000 people aged 50–64 years to 4.91 per 100,000 people aged 65–74 years, and 8.69 per 100,000 people over 75 years [3]. In this study, the mean age at diagnosis was 71.2 ± 12.6 years, mirroring previous studies with a mean age of 64–69.4 years [2, 3, 11].

Female gender increases the risk of GBC by twofold to sixfold [12]. In the current study, GBC was significantly more common among females with a female-to-male ratio of 2.41 : 1. Although, female gender is associated with a higher risk of GBC, its effect varies based on ethnicity and geographies [1]. The female-to-male ratio of GBC has been reported to be as high as 3.0 : 1 among Hispanics, compared to 1.28 : 1 in African American populations [1]. Early studies on GBC have also noted increased incidence in multiparous females, suggesting a link between GBC and hormone levels [8]. Further studies have found estrogen and progestin receptors in GBC [3].

The most significant risk factor for GBC is gallstones (relative risk (RR) = 3.0–23.8) and is present in the majority (69–85%) of patients [3, 13]. Stones irritate gallbladder mucosa resulting in inflammation, which may eventually results in carcinogenesis [3]. Patients with chronic gallstones, defined as present for 20 or more years, have an increased risk of GBC (OR = 6.2–12.1) [3]. Stone size has also been shown to contribute to an increased risk of GBC, with stones larger than 3 cm increasing risk by over ninefold (RR = 9.2–10.1) [3].

The prognosis of GBC is extremely poor, most often due to its late diagnosis. Patients with GBC in this study often had advanced stage and grade by the time of diagnosis. The majority of patients presented with either poorly (42.5%) or moderately (38.2%) differentiated disease, and over 85% of patients had lymph node involvement.

The extensive progression of disease can be explained at least in part due to the difficulty in diagnosis [2]. Patients with GBC frequently have nonspecific symptoms such as vague abdominal pain and discomfort [3]. Common complaints include constitutional symptoms, anorexia, and weight loss progressing to painless jaundice [3, 14]. Furthermore, disease progression occurs silently over years [15].

GBC most commonly arises due to the dysplasia-carcinoma sequence but can occasionally occur due to polyps and adenoma-carcinoma progression [1, 14, 16]. Progression from metaplasia to dysplasia may require up to ten years and the development of carcinoma in situ an additional five years [1]. Metaplasia most commonly arises due to inflammation caused by chronic gallstone irritation [14]. Inflammation then leads to increased expression of COX2 and inhibition of tumor suppressor genes such as p53 [14, 17, 18].

The near silent and chronic progression of GBC results in many diagnoses (70%) detected incidentally [2]. Patients with GBC are commonly operated upon for diseases such as gallstones, cholecystitis, or polyps and the cancer is discovered incidentally with a frequency of 0.2% to 3.0% of all cholecystectomies [2, 3]. The recent adoption of laparoscopic surgeries has led to an increase in the frequency of cholecystectomies and therefore an increase in GBC diagnosis [2, 19–22]. This is most apparent in this study in the years between 2000 and 2010, as the number of cholecystectomies and surgical resections doubled.

The tragic consequence of incidental discovery and late diagnosis is a one-year survival of 34%, a cumulative five-year survival of 13%, and a mean overall survival of only 2.7 years. Previous studies have specifically recognized extended disease and the number of positive lymph nodes as important predictors of worsening outcomes [23]. Factors associated with extended disease such as moderately and poorly differentiated tumors are independently associated with increased mortality for GBC.

Surgical resection is the standard of care for GBC patients [24]. In localized disease, simple cholecystectomy may be sufficient, and several studies have demonstrated similar survival with cholecystectomy compared to more radical extended resections [24–27]. In advance disease however, reresection after a simple cholecystectomy or radical resection is associated with significantly improved mortality [25, 28].

The use of radiation as a treatment modality alone has inferior survival rates compared to surgical resection and is typically used in combination with chemotherapy when surgery is not feasible [29]. In the current study, patients undergoing radiation alone survived 9.8 months compared to 3.7 years with surgical resection. The addition of radiation to surgical therapy improved overall survival by a little more than 3 months (4.0 versus 3.7 years with surgical resection alone). Hoehn et al. (2015) conducted a study involving 6,690 GBC patients from the American College of Surgeons National Cancer Data Base and reported that adjuvant chemoradiation significantly improved survival (Hazard Ratio (HR) = 0.77; 95% CI, 0.66–0.90), while adjuvant chemotherapy did not affect survival [30].

Future improvements in therapy are focused on individual processes of carcinogenesis [14]. Current studies are investigating use of small molecule pathway inhibitors and monoclonal antibodies [3, 14, 24]. Several small clinical trials utilizing monoclonal antibodies targeting epidermal growth factor receptor (EGFR), vascular endothelial growth factor receptor (VEGFR), human epidermal growth factor receptor 2 (HER2), and multikinase inhibitors such as sorafenib have been completed or are on-going with demonstrable evidence of treatment effect and improved survival [14].

There are several limitations to this study which need to be considered. The SEER database does not accurately code all clinical factors which may affect patient survival. Secondly, information on chemotherapy received was not provided in detail, limiting this study's ability to evaluate the effect of adjuvant or neoadjuvant therapy. There may also be an element of selection bias, since SEER registries are more likely to sample from urban than from rural areas. Despite these limitations, the SEER database has data obtained a representative sample of the US population and therefore these findings can be generalized to the overall population.

## 5. Conclusions

GBC is an uncommon malignancy that presents most often among females in their 8th decade of life, with advanced stage of disease and lymph node involvement. The incidence of GBC has doubled in the last decade concurrent with increases in cholecystectomy rates attributable in part to improved histopathological detection, as well as laparoscopic advances and enhanced endoscopic techniques. Surgical resection confers significant survival benefit in GBC patients. The role of radiation therapy remains controversial, and adjuvant radiation therapy in addition to surgical resection has been shown to confer a small survival advantage. Despite treatment, overall and cancer-specific survival remains low. Given its rarity, all GBC patients should be enrolled in clinical trials or registries to optimize treatment and clinical outcomes for these patients.

## Figures and Tables

**Table 1:** Annual cases of gallbladder carcinoma from the Surveillance, Epidemiology, and End Result (SEER) database (1973–2013).

Year	New cases
1973	246
1974	289
1975	304
1976	261
1977	294
1978	299
1979	306
1980	331
1981	332
1982	289
1983	294
1984	325
1985	296
1986	287
1987	294
1988	285
1989	304
1990	294
1991	292
1992	447
1993	419
1994	407
1995	449
1996	397
1997	414
1998	422
1999	426
2000	820
2001	886
2002	837
2003	924
2004	874
2005	937
2006	935
2007	975
2008	907
2009	1,002
2010	1,009
2011	1,042
2012	1,089
2013	1,103

**Table 2:** Demographics and clinical profile of 22,343 patients with gallbladder carcinoma from the Surveillance, Epidemiology, and End Result (SEER) database (1973–2013).

Variables	Frequency (%)
*N*	22,343
*Age*, years (mean ± SD)	71.17 ± 12.534
*Gender*, *N* (%)	
Male	6,549 (29.3%)
Female	15,794 (70.7%)
*Region*, *N* (%)	
Alaska	46 (0.2%)
East	6,684 (29.9%)
Northern Plains	4,230 (18.9%)
Pacific Coast	9,605 (43.0%)
Southwest	1,778 (8.0%)
*Race*, *N* (%)^**∗∗**^	
Caucasian	14,280 (64.0%)
African American	2,056 (9.2%)
Hispanic	3,740 (16.8%)
Asian/Pacific Islander	1,861 (8.3%)
American Indian/Alaska Native	375 (1.7%)
*Grade*, *N* (%)^*∗∗*^	
Well differentiated	2,252 (15.3%)
Moderately differentiated	5,619 (38.2%)
Poorly differentiated	6,238 (42.5%)
Undifferentiated	592 (4.0%)
*Lymph node involvement*, *N* (%)^*∗∗*^	
Yes	15,791 (88.2%)
No	2,105 (11.8%)
*Treatment received*, *N* (%)^*∗∗*^	
No treatment	6,811 (31.8%)
Surgery only	11,769 (55.0%)
Radiation only	545 (2.6%)
Both surgery and radiation	2,269 (10.6%)
*Actuarial survival*, years (mean ± SE)	2.715 ± 0.061
*Actuarial survival by treatment*, years (mean ± SE)	
No treatment	0.618 ± 0.049
Surgery only	3.685 ± 0.093
Radiation only	0.815 ± 0.075
Both surgery and radiation	4.029 ± 0.184
*Actuarial survival by grade*, years (mean ± SE)	
Well differentiated	5.926 ± 0.266
Moderately differentiated	3.720 ± 0.151
Poorly differentiated	1.664 ± 0.073
Undifferentiated	1.293 ± 0.167
*Overall mortality*, *N* (%)	19,439 (87.0%)
*Cancer specific mortality*, *N* (%)	16,856 (75.4%)
*Cumulative survival*, %	
3-month	66%
6-month	50%
9-month	41%
1-year	34%
2-year	22%
3-year	17%
4-year	14%
5-year	13%

*N* = number; SD = standard deviation; SE = standard error; ^*∗∗*^data presented for patients with available information only.

**Table 3:** Survival outcomes of 22,343 patients with gallbladder carcinoma from the Surveillance, Epidemiology, and End Result (SEER) database (1973–2013).

	Overall	Surgery alone	Radiation alone	Both surgery and radiation	Neither
*Actuarial survival by treatment*, years (mean ± SE)	2.715 ± 0.061	3.685 ± 0.093	0.815 ± 0.075	4.029 ± 0.184	0.618 ± 0.049

*Cumulative survival, %*					
3-month	66%	75%	76%	96%	42%
6-month	50%	61%	50%	84%	23%
9-month	41%	51%	32%	72%	14%
1-year	34%	44%	29%	60%	10%
2-year	22%	31%	21%	35%	5%
3-year	17%	24%	6%	26%	3%
4-year	14%	21%	3%	21%	2%
5-year	13%	18%	2%	18%	2%

SE = standard error.
